# The monoaminergic footprint of depression and psychosis in dementia with Lewy bodies compared to Alzheimer’s disease

**DOI:** 10.1186/s13195-014-0090-1

**Published:** 2015-02-11

**Authors:** Yannick Vermeiren, Debby Van Dam, Tony Aerts, Sebastiaan Engelborghs, Jean-Jacques Martin, Peter P De Deyn

**Affiliations:** Laboratory of Neurochemistry and Behavior, Institute Born-Bunge, University of Antwerp, Campus Drie Eiken, Universiteitsplein 1, Wilrijk, Antwerp, 2610 Belgium; Department of Neurology and Memory Clinic, Hospital Network Antwerp (ZNA) Middelheim and Hoge Beuken, Lindendreef 1, Antwerp, 2020 Belgium; Department of Neurology and Alzheimer Research Center, University of Groningen, University Medical Center Groningen (UMCG), Hanzeplein 1, Groningen, 9713, GZ The Netherlands; Biobank, Institute Born-Bunge, University of Antwerp, Universiteitsplein 1, Antwerp, 2610 Belgium

## Abstract

**Introduction:**

Depression and psychosis are two of the most severe neuropsychiatric symptoms (NPS) in dementia with Lewy bodies (DLB) and Alzheimer’s disease (AD). Both NPS have negative effects on cognitive performance and life expectancy. The current study aimed to investigate and compare monoaminergic etiologies between both neurodegenerative conditions, given the lack of an efficient pharmacological treatment until present.

**Methods:**

Eleven behaviorally relevant brain regions of the left frozen hemisphere of 10 neuropathologically confirmed AD patients with/without depression (AD + D/-D; 5 were psychotic within AD + D), 10 confirmed DLB patients, all of whom were depressed (DLB + D; 5 psychotic patients), and, finally, 10 confirmed control subjects were regionally dissected. All patients were retrospectively assessed before death using the *Behavioral Pathology in Alzheimer’s Disease Rating Scale* (Behave-AD) and *Cornell Scale for Depression in Dementia* amongst others. The concentrations of dopamine (DA), serotonin (5-HT), (nor)adrenaline and respective metabolites, i.e. 3,4-dihydroxyphenylacetic acid (DOPAC) and homovanillic acid (HVA), 5-hydroxy-3-indoleacetic acid (5-HIAA), and, 3-methoxy-4-hydroxyphenylglycol (MHPG), were determined using reversed-phase high-performance liquid chromatography with electrochemical detection.

**Results:**

DLB subjects had the overall lowest monoamine and metabolite concentrations regarding 33 out of 41 significant monoaminergic group alterations. Moreover, MHPG levels were significantly decreased in almost 8 out of 11 brain regions of DLB- compared to AD patients. We also observed the lowest 5-HT and 5-HIAA levels, and 5-HIAA/5-HT turnover ratios in DLB + D compared to AD + D subjects. Additionally, a 4- and 7-fold increase of DOPAC/DA and HVA/DA turnover ratios, and, a 10-fold decrease of thalamic DA levels in DLB + D compared to AD + D patients and control subjects was noticed. Regarding psychosis, hippocampal DA levels in the overall DLB group significantly correlated with Behave-AD AB scores. In the total AD group, DA levels and HVA/DA ratios in the amygdala significantly correlated with Behave-AD AB scores instead.

**Conclusions:**

Monoaminergic neurotransmitter alterations contribute differently to the pathophysiology of depression and psychosis in DLB as opposed to AD, with a severely decreased serotonergic neurotransmission as the main monoaminergic etiology of depression in DLB. Similarly, psychosis in DLB might, in part, be etiologically explained by dopaminergic alterations in the hippocampus, whereas in AD, the amygdala might be involved.

## Introduction

Dementia with Lewy bodies (DLB) is the second most common neurodegenerative disorder following Alzheimer’s disease (AD) and accounts for up to 20% of all autopsy-confirmed dementias in the elderly [1,2]. One of the key hallmarks of DLB patients, besides the cognitive impairment and parkinsonian symptomatology, is the high frequency of neuropsychiatric symptoms (NPS), particularly psychosis [3]. The presence of recurrent visual hallucinations has even been identified as one of the core features in the clinical diagnosis of DLB. Additionally, the supportive and suggestive diagnostic features include depression, systematized delusions and rapid eye movement (REM) sleep behavior disorders among others [4]. Typically, visual hallucinations, delusions and depression are much more common in DLB than in AD [5,6]. The first two NPS occur in approximately 60% to 70% of DLB patients [5-7], whereas depression, although frequently present in AD (28%), remains much more persistent in DLB (45%) after a follow-up period of one year [8]. Moreover, depressive symptoms in AD and DLB are associated with a greater cognitive decline [8] and, in AD, significantly relate to lower survival rates over a three-year period [9]. Psychosis in AD is also very common and has previously been associated with an increased mortality rate and, again, an accelerated cognitive decline [10,11]. Besides depression and psychosis, symptoms of anxiety, apathy and sleep disturbances often coexist in DLB patients [3,12]. In addition, delusions and hallucinations may trigger other NPS, such as agitation or aggression, which regularly leads to early nursing home admission [12].

At present, regarding the different classes of psychoactive drug therapies to alleviate depression and psychosis in AD, antipsychotics are the primary pharmacological treatment option, although they may induce serious side effects, increase mortality rates [13], and their efficacy is ‘modest’ at best [14]. The administration of psychotropic medication has also been associated with a more rapid cognitive and functional decline, and not necessarily with improved NPS [15]. As for antidepressants, Pollock and colleagues [16] reported that citalopram was superior to placebo, with greatest efficacy for aggression, and, in a later study [17], citalopram was found to be comparable in efficacy to risperidone, differentiated by its significant effect on agitation and its superior tolerability in the treatment of moderate to severe NPS. Randomized controlled trials of sertraline [18] and trazodone [19], however, have been less promising. The pharmacological treatment of NPS in DLB patients, on the other hand, requires an even more cautious approach. For example, all drugs with anticholinergic side effects, such as tricyclic antidepressants, low potency neuroleptics, antiparkinsonian anticholinergic drugs and antispasmodics for bladder or gastrointestinal tract, should be avoided due to their potential to exacerbate psychotic symptoms and, moreover, might induce orthostatic hypotension [12]. Consequently, cholinomimetic therapy using cholinesterase inhibitors has proven to be beneficial for apathy, anxiety and psychosis [20-22], while two other studies found weak [23] or unclear [24] evidence to support its use in DLB compared to Parkinson’s disease dementia (PDD). Interestingly, concurrent treatment with a selective serotonin reuptake inhibitor (SSRI) and a serotonin (5-hydroxytryptamine, 5-HT) 1A receptor antagonist might offer a positive outcome to treat depression efficiently in DLB, as was suggested by Francis [25], although evidence for the benefits of antipsychotics other than clozapine is limited, and there are serious safety concerns about the use of antipsychotics in these patients [3]. Finally, the administration of memantine, an N-methyl-D-aspartate (NMDA) receptor antagonist, may result in variable symptomatic side effects in DLB patients, including worsening of psychosis or even an adverse drug reaction [26,27].

While the neurobiological aspects of the parkinsonism of DLB patients have been intensively studied, much less attention has been paid to the pathophysiological mechanisms underlying depression and psychosis associated with DLB. More specifically, very few studies have attempted to delineate their neurochemical correlates [25,28-31] and whether these are similar to or distinct from AD [25]. Furthermore, given the lack of an efficient psychotropic monoaminergic therapy, and due to the fact that psychosis and depression are enormously troublesome to caregivers and patients, cause an earlier institutionalization and account for a significant increase in the overall cost of dementia, it becomes essential to intensely investigate the monoaminergic pathophysiology of NPS features in DLB compared to AD. Likewise, although recently a handful of studies have examined the monoaminergic etiology of NPS in AD, as well as in DLB and frontotemporal dementia, in postmortem brain tissue [32] and cerebrospinal fluid (CSF) samples [33], a neurochemical comparison study between DLB and AD supplemented with the inclusion of an age- and gender-matched control group would give more insight into the monoaminergic characteristics of depression and psychosis of both neurodegenerative disorders separately. In the long term, such comparison studies may contribute to the development of novel psychotropic pharmacotherapies [3,34].

We, therefore, determined the levels of eight monoamines and metabolites, that is, the indolamine 5-HT, the catecholamines dopamine (DA), adrenaline (A) and noradrenaline (NA), as well as their respective metabolites, that is, 5-hydroxy-3-indoleacetic acid (5-HIAA; metabolite of the serotonergic neurotransmitter system), 3,4-dihydroxyphenylacetic acid and homovanillic acid (DOPAC and HVA, respectively; metabolites of the dopaminergic neurotransmitter system) and 3-methoxy-4-hydroxyphenylglycol (MHPG; metabolite of the (nor)adrenergic neurotransmitter system) in various postmortem brain regions of depressed and/or psychotic DLB and AD patients using reversed-phase high-performance liquid chromatography with electrochemical detection (RP-HPLC-ECD) to identify monoaminergic neurotransmitter alterations which may underlie both NPS. As a baseline reference, tissue samples of the same brain regions of a healthy control group were neurochemically analyzed as well.

## Methods

### Study population and inclusion protocol

Neuropathologically confirmed AD patients with (n = 10; AD + D) and without (n = 10; AD-D) depression, 10 confirmed DLB patients, all of whom were depressed (DLB + D) and, finally, 10 confirmed control subjects were selected from the Biobank of the Institute Born-Bunge (University of Antwerp, Antwerp, Belgium). Initially, all patients with a clinical diagnosis of *probable* AD and DLB were recruited at the Memory Clinic of the Hospital Network Antwerp (ZNA-Middelheim and ZNA-Hoge Beuken, Antwerp, Belgium) for inclusion in a prospective, longitudinal study on NPS [35]. As part of their differential diagnostic work-up of dementia, besides the general physical and neurological examinations, blood screening tests, structural neuroimaging by computed tomography (CT), magnetic resonance imaging (MRI) or single photon emission computed tomography (SPECT), neuropsychological evaluations (for example, Mini-Mental State Examination (MMSE) scores) and optional cerebrospinal fluid (CSF)/blood sampling for biomarker and/or DNA analyses, a baseline behavioral assessment was routinely performed. If possible, AD and DLB patients were behaviorally rated again during follow-up. The clinical diagnosis of *probable* AD was based on the NINCDS/ADRDA criteria of McKhann *et al*. [36,37] whereas *probable* DLB was diagnosed according to the consensus guidelines of McKeith *et al*. [4,38]. All patients also fulfilled the *Diagnostic and Statistical Manual of Mental Disorders – IV – text revision* (DMS-IV-TR) criteria [39]. On the other hand, age-matched control subjects were hospitalized in the Middelheim General Hospital (Antwerp, Belgium) and consented shortly before death. Death causes were cardiac failure due to an acute myocardial infarct (n = 2), chronic obstructive pulmonary disease (COPD) (n = 3), carcinoma (hepatic (n = 1); prostate (n = 1); lung (n = 1); neuroendocrine (n = 1)) and multiple myeloma (n = 1). Moreover, clinical neurological examination, as well as a retrospective review of the clinical history, neuropsychological evaluation and hospital records, did not reveal any evidence of dementia, psychiatric antecedents or cognitive decline. Written informed consent regarding autopsy and the subsequent use of brain tissue, clinical documentation and behavioral data for research purposes was obtained from all participants. The study was also approved by the Medical Ethical Committee of the Middelheim General Hospital (Antwerp, Belgium) and conducted in compliance with the Helsinki Declaration.

In case consented AD, DLB or control subjects died, brain autopsy was performed within six hours after death after which the left hemisphere was frozen at −80°C for neurochemical analyses, and the right hemisphere was formaldehyde-fixated for neuropathological examination. The 10 AD + D and 10 AD-D patients were part of a larger cohort of 40 behaviorally characterized AD patients who were previously subjected to RP-HPLC-ECD assessments [32]. However, the inclusion of DLB patients and control subjects, as well as the inclusion of several extra brain regions, render the current study significantly distinct from our previous one [32], which had the same methodological setup, but an entirely different hypothesis.

None of the included control subjects suffered from central nervous system pathology.

### Behavioral assessment

Behavior of AD and DLB patients was assessed together with relatives and/or nursing staff using a battery of behavioral assessment scales, including: the Behavioral Pathology in Alzheimer’s Disease Rating Scale (Behave-AD) [40]; Middelheim Frontality Score (MFS) [41]; Cohen-Mansfield Agitation Inventory (CMAI) [42]; and Cornell Scale for Depression in Dementia (CSDD) [43]. Dementia staging was based on the Global Deterioration Scale (GDS) with a range varying from 1 (nondemented) to 7 (terminal stage of dementia) [44]. During each NPS assessment, only the behavioral phenomena covering the last two weeks prior to the assessment were included and rated. Behavioral assessment was repeated during each neurological follow-up examination in the hospital, if possible (n = 2 for AD + D, n = 3 for AD-D and n = 6 for DLB + D). A final retrospective behavioral scoring was performed in case patients died more than ten days after the last follow-up or baseline rating. In total, eight AD + D-, seven AD-D- and four DLB + D patients underwent only one rating close to death, given the short amount of time which was left since they entered our study protocol. Nevertheless, for this research purpose, only the final behavioral assessment scores around the date of death were used so that possible neurochemical alterations in the brain were as representative as possible for the concurrent clinical manifestation of NPS in all dementia patients.

Patients with a total CSDD cutoff score ≥8 were classified as depressed, whereas patients with a CSDD score <8 were defined as non-depressed [45]. Furthermore, patients with a Behave-AD cluster A score (delusions) ≥4 *or* a Behave-AD cluster B score (hallucinations) ≥2 *or* patients who were rated on the Behave-AD cluster A *and* B subscales (delusions + hallucinations), irrespective of its combined value, were classified as psychotic [46]. Finally, based on their behavioral profiles, AD and DLB patients were divided into three main groups, that is, AD + D (n = 10, of which 5 were psychotic (AD + D + P), AD-D (n = 10, of which none were psychotic (AD-D-P)) and DLB + D (n = 10, of which 5 were psychotic (DLB + D + P).

No behavioral scores were available for the control group.

### Neuropathological examination and criteria

In order to neuropathologically confirm or reject the clinically established AD and DLB diagnoses, immunohistochemistry on a standard selection of 10 to 13 regionally dissected postmortem brain regions of the formaldehyde-fixated right hemisphere was performed [47]. In short, the stains routinely applied in our laboratory on paraffin blocks of the neocortex (frontal, temporal and occipital), amygdala, rhinencephalon (at the level of the posterior part of the amygdala and the lateral geniculate body (for hippocampus)), basal ganglia, thalamus, brain stem, substantia nigra (SN), pons at the level of the locus coeruleus (LC) and cerebellum are hematoxylin-eosin (HE), cresyl violet and Bodian’s technique. The routinely applied immunostains are 4G8 (amyloid), AT8 (P-tau_181P_) and an anti-ubiquitin antibody (ubiquitin). When the presence of Lewy bodies (LB) is suspected on HE staining and ubiquitin immunoreactivity, an additional anti-α-synuclein staining is applied.

Neuropathological assessments were established by the same neuropathologist (JJM), employing the criteria of Braak and Braak [48], Braak *et al*. [49] and Jellinger and Bancher [50] to decide on *definite* AD. The ‘ABC scoring’ method of Montine *et al*. [51] was applied to all AD brains collected after May 2011 (n = 5). Similarly, the consensus guidelines of McKeith *et al*. [4,38] were used for the neuropathological diagnosis of *definite* DLB. In case of significant concurrent AD pathology (n = 8 out of 10 DLB patients), the likelihood that the pathological findings were associated with a DLB clinical syndrome was assessed as recommended by McKeith *et al*. [4], resulting in a final decision of *definite* DLB with limbic translational (n = 4; including n = 2 without AD pathology) and diffuse neocortical (n = 6) Lewy body subtype pathologies.

Regarding the control subjects, microscopic examination failed to detect significant degenerative changes in any of the control brains, except for a limited cerebral amyloid angiopathy, if present at all, and the sporadic presence of some neurofibrillary (pre)tangles or diffuse senile plaques, all of which were considered age-related.

### Regional brain dissection

Regional brain dissection of the left frozen hemisphere was performed according to a standard procedure during which 21 brain regions were routinely dissected. With regard to this specific study design, a total of 11 behaviorally and neurochemically relevant brain areas were ultimately analyzed using RP-HPLC-ECD, that is, Brodmann area (BA) 9 and BA10 (prefrontal cortex), BA11 (orbitofrontal cortex), BA17 (occipital cortex), BA22 (temporal cortex), BA24 (anterior cingulate gyrus), amygdala, hippocampus, thalamus, cerebellar cortex and LC. All of these brain regions have previously been the subject of other NPS-related neurochemical research in depressed and/or psychotic AD and DLB patients [25,28-31,52-56] and, moreover, are also part of integrated behavior-modifying brain circuits, such as the dopaminergic mesolimbic system among others. In addition, this careful selection comprises regions with clear-cut AD and DLB neuro-pathological and -imaging hallmarks (for example, BA22, hippocampus, prefrontal areas, thalamus and LC), as well as brain regions in which these lesions are less present to absent (BA17 and cerebellar cortex), but might still hold significant neurochemical alterations.

Dissections were performed on a plastic cutting board above a mixture of regular and dry ice by means of scalpel, tweezers and a Dremel® 200 series rotary instrument tool according to a standard procedure. During the first part of this protocol, 15 of the 21 brain regions were meticulously dissected as they were easily accessible, that is, BA6/7/8/9/10/11/12/17/22/24/46, SN, cerebellar cortex, medulla oblongata and LC. Afterwards, the remaining left hemisphere was placed at −20°C for four hours after which seven to eight coronal slices were cut. Subsequently, the remaining six brain regions were dissected, namely amygdala, hippocampus, thalamus, caudate nucleus, putamen and globus pallidus. All dissected brain tissue samples roughly weighed 300 to 500 mg and were immediately stored on dry ice in precooled aluminum cryovials (Sanbio BV, Uden, The Netherlands) during the dissection. In total, each dissected brain region could be subdivided into four of these cryovials of which three were used for HPLC analyses. The fourth one was used for pH measurement if necessary.

All AD, DLB and control brain hemispheres were dissected by the same researcher to minimize variability.

### Neurochemical RP-HPLC-ECD analysis

A recently optimized and validated RP-HPLC system with electrochemical detection (ECD) for the fast detection of monoaminergic compounds in human brain tissue was used to simultaneously measure the concentrations of 5-HT, DA, (N)A and their respective metabolites, that is, 5-HIAA, DOPAC/HVA and MHPG [57]. Sample analysis was performed using an Alexys^TM^ Dual Monoamines Analyzer (Antec Leyden BV, Zoeterwoude, The Netherlands) by which each brain tissue sample was directly analyzed in duplicate. Output ranges were 500 pA and 2 nA, respectively, with two electrochemical VT03 flow cells each containing a glassy carbon working electrode of 0.7 mm and an *in situ* Ag/AgCl reference electrode at 670 mV placed in a Decade II electrochemical detector (Antec Leyden BV, Zoeterwoude, The Netherlands). An isocratic flow rate of 40 μL of mobile phase per minute was set for both LC 110 pumps. The optimal conditions for separation of the monoaminergic compounds were obtained using a mobile phase comprising 13% methanol combined with a mixture of phosphoric (50 mM) and citric acid (50 mM), octane-1-sulfonic acid sodium salt (1.8 mM), KCl (8 mM) and ethylenediaminetetraacetic acid (EDTA) (0.1 mM) (pH 3.6). Samples were loaded onto two microbore ALF-125 columns (250 mm × 1.0 mm, 3 μm particle size) filled with a porous C18 silica stationary phase by an Alexys^TM^ AS 100 Autosampler (Antec Leyden BV, Zoeterwoude, The Netherlands). Separation of the monoamines and metabolites was achieved at a stable column and VT03 flow cell temperature of 36°C with a total runtime of only 45 minutes per sample. Levels of the monoaminergic compounds were calculated using Clarity^TM^ Software (DataApex Ltd, 2008, Prague, The Czech Republic). All chemicals were of analytical grade.

The brain sample preparation procedure prior to RP-HPLC-ECD analysis was simple and fast. Initially, 200 to 300 mg frozen brain tissue was weighed in 4 ml mobile phase. Next, this mixture was homogenized for 40 seconds (50 seconds in case samples that weighed ≥250 mg) at moderate speed using an Ultra-Turrax TR 50 homogenizer® (Janke & Kunkel, Ika-Werk, Staufen, Germany). The homogenate was then centrifuged (26,000 × g, 20 minutes, 4°C) and afterwards, the supernatant was filtered using a 0.2 μm Millipore® filter (Millex, Merck Millipore, Carrigtwohill, County Cork, Ireland) attached to a syringe. Further elimination of proteins was accomplished using 10 kDa Amicon® Ultra Centrifugal Filters (Millipore) (14,000 × g, 20 minutes, 4°C) which were washed twice beforehand with mobile phase. The final filtrate was then loaded onto the HPLC columns (undiluted and diluted).

### pH measurement of cerebellar brain tissue

Samples needed to be nonacidotic (that is, pH >6.1) [58,59] in order to guarantee high-quality brain tissue since acidosis may induce alterations in neurotransmitter and neuropeptide concentrations, as well as enzyme activity. Several factors such as a prolonged death struggle, premortem stroke and a long postmortem delay could acidify brain tissue [60,61]. In this study, pH values of the cerebellar cortex were measured since the cerebellar pH has previously been shown to be most representative for the entire brain [61].

First, a 0.01 N NaOH-solution was prepared to adjust the pH of deionized water to 7.0. Next, approximately 100 mg of frozen cerebellar cortex was weighed after which 1 ml of the adjusted water was added. The mixture was then homogenized with a Pro-200 Homogenizer (Pro Scientific, Oxford, CT, USA) for 30 seconds at moderate speed at 4°C. Finally, the homogenate was centrifuged (8,000 × g, 10 minutes, 4°C) and the pH of the supernatant was measured. Per patient, two cerebellar samples were included.

At the end, all brain regions with acidotic pH values (<6.1) were excluded from data analysis.

### Statistical analysis

Nonparametric statistics were applied due to the limited number of patients, ordinal variables (behavioral scores) and not normally distributed data after performing a Shapiro-Wilk test of normality.

Chi-square statistics were applied to compare male/female ratios and patients taking/not taking psychotropic medication across groups. Kruskal-Wallis analyses with *post-hoc* Mann-Whitney U tests were used for comparison of all behavioral, neurocognitive, demographic, pH and monoaminergic data between AD + D, AD-D, DLB + D and control subjects, as well as the monoaminergic data between AD + D + P, AD + D-P, DLB + D + P and DLB + D-P. In all cases, only data remaining statistically significant following a Bonferroni correction for multiple comparisons (*P* <0.017 for three group comparisons (Table 1) and *P* <0.0083 for six group comparisons (Tables 2 and 3)) were regarded as significant. Mann–Whitney U tests were used to look at potential confounding effects of psychotropic medication, with regard to our neurochemical data, between patients taking and not taking psychotropic medication within each group. Finally, in order to calculate neurochemical correlations of CSDD-, Behave-AD cluster A/B/A + B- and other behavioral scores in the total AD (n = 20) and DLB (n = 10) populations, nonparametric Spearman’s Rank-Order correlation statistics were applied. Again, a Bonferroni correction was performed, and only those significant data were taken into account (Table 4; *P* <0.0033). All analyses were performed using SPSS 22.0 for Windows (IBM SPSS Software, Armonk, NY, USA, IBM Corp.). Figures were generated using GraphPad Prism 6.0 software.

**Table 1:** **Clinical data, behavioral assessment scores and pH values**

**Parameter**	**AD + D (number = 10)**	**AD-D (number = 10)**	**DLB + D (number = 10)**	**CONTR (number = 10)**	**Kruskal Wallis**
**Demographics and clinical data**					
Age at onset dementia (years)	71.5 ± 14.0 (53–94)	79.4 ± 13.5 (50–96)	76.0 ± 5.8 (66–84)	N/A	*P* = 0.286
Age at death (years)	75.7 ± 12.3 (58–95)	83.5 ± 11.6 (59–97)	81.4 ± 4.4 (72–87)	76.5 ± 7.0 (65–87)	*P* = 0.148
Gender, male/female (number)	6/4	7/3	7/3	5/5	χ^2^ = 1.173; *P* = 0.759
Storage time tissue at −80°C (years)	4.3 ± 3.8 (0.3-10)	4.1 ± 4.0 (0.6-10)	6.6 ± 4.3 (2–11)	8.6 ± 1.0 (7–10)	*P* = 0.060
Postmortem delay^c^ (hours)	3.7 ± 1.0 (2–5)	3.7 ± 0.9 (2–6)	4.2 ± 1.4 (2–6)	4.8 ± 1.6 (3–6)	*P* = 0.384
Taking/not taking psychotropic medication (number)	5/5	7/3	6/4	4/6	χ^2^ = 1.125; *P* = 0.771
**Behavioral assessment scores**					
MFS total score (/10)	4.8 ± 0.9 (3–6)^aa^	2.3 ± 1.8 (1–5)^aa^	4.3 ± 1.3 (3–7)	N/A	*P* = 0.008
Behave-AD cluster A (/21)	1.4 ± 2.4 (0–6)	0.10 ± 0.3 (0–1)	0.4 ± 0.8 (0–2)	N/A	*P* = 0.401
Paranoid and delusional ideation					
Behave-AD cluster B (/15)	0.8 ± 1.2 (0–3)	0.0 ± 0.0^b^	1.9 ± 2.2 (0–6)^b^	N/A	*P* = 0.033
hallucinations					
Behave-AD cluster AB (/36)	2.2 ± 2.5 (0–6)	0.1 ± 0.3 (0–1)^b^	2.3 ± 2.3 (0–6)^b^	N/A	*P* = 0.041
psychosis					
Behave-AD cluster C score (/9)	2.9 ± 2.8 (0–7)	0.5 ± 0.9 (0–2)	0.9 ± 1.3 (0–4)	N/A	*P* = 0.084
activity disturbances					
Behave-AD cluster D score (/9)	4.5 ± 2.9 (0–9)	1.7 ± 1.9 (0–4)	1.8 ± 2.4 (0–7)	N/A	*P* = 0.052
aggressiveness					
Behave-AD cluster E score (/3)	0.7 ± 0.8 (0–2)	0.1 ± 0.3 (0–1)	0.3 ± 0.7 (0–2)	N/A	*P* = 0.111
diurnal rhythm disturbances					
Behave-AD cluster F score (/6)	1.9 ± 1.7 (0–4)	0.3 ± 0.7 (0–2)	1.1 ± 1.4 (0–4)	N/A	*P* = 0.052
affective disturbances					
Behave-AD cluster G score (/12)	1.2 ± 2.0 (0–5)	0.0 ± 0.0	0.8 ± 1.7 (0–4)	N/A	*P* = 0.200
anxieties and phobias					
Behave-AD total score (/75)	13.4 ± 8.9 (2–24)^aa^	2.7 ± 2.4 (0–6)^aa^	7.3 ± 5.3 (2–17)	N/A	*P* = 0.009
Behave-AD global score (/3)	1.7 ± 0.9 (0–3)^aa^	0.4 ± 0.5 (0–1)^aa^	1.2 ± 0.8 (0–2)	N/A	*P* = 0.006
caregiver burden					
CMAI cluster 1 (/70)	15.4 ± 8.1 (10–35)	10.8 ± 2.5 (10–18)	11.1 ± 2.4 (10–17)	N/A	*P* = 0.041
aggressive behavior					
CMAI cluster 2 (/77)	24.7 ± 10.9 (11–39)^a^	13.1 ± 3.3 (11–21)^a^	18.5 ± 7.5 (11–33)	N/A	*P* = 0.021
physically nonaggressive behavior					
CMAI cluster 3 (/56)	16.1 ± 8.6 (8–33)	11.8 ± 5.6 (8–23)	14.9 ± 7.6 (8–25)	N/A	*P* = 0.344
verbally agitated behavior					
CMAI total sore (/203)	56.2 ± 22.1 (29–90)	35.7 ± 7.4 (29–47)	44.5 ± 12.3 (29–65)	N/A	*P* = 0.040
CSDD total score (/38)	12.1 ± 3.3 (8–19) ^aaa^	5.0 ± 1.2 (3–7) ^aaa,^ ^bbb^	10.1 ± 2.1 (8–14)^bbb^	N/A	*P* <0.0001
depression					
Interval between rating and death (days)	3.8 ± 10.1 (0–6)	3.9 ± 7.0 (0–9)	5.2 ± 9.4 (0–10)	N/A	*P* = 0.713
**MMSE, staging and pH data**					
MMSE score^d^ (/30)	12.0 ± 6.6 (7–21) number = 4	16.0 ± 4.3 (10–25)^b^ number = 8	8.7 ± 4.8 (4–16)^b^ number = 6	N/A	*P* = 0.041
GDS score: dementia staging (/7)	6.2 ± 0.8 (5–7)	5.8 ± 1.0 (4–7)	6.6 ± 0.5 (6–7)	N/A	*P* = 0.151
pH values cerebellar brain tissue	6.5 ± 0.3 (5.9-7.1)	6.5 ± 0.3 (6.0-6.8)	6.7 ± 0.3 (6.4-7.2)	6.4 ± 0.2 (6.0-6.7)	*P* = 0.300

Mean ± SD with minimum-maximum ranges between parentheses; Kruskal-Wallis analyses (*P* <0.05) with *post-hoc* Mann-Whitney U tests were performed; data remaining statistically significant following Bonferroni correction are presented above with one superscript letter (*P* <0.017; for three groups comparisons because CONTR data are absent); χ^2^ statistics were used to compare male/female ratios and taking/not taking any type of psychotropic medication; significant differences with *P* <0.01 and *P* <0.0001 are respectively indicated with two and three repeated superscript letters; the following letters are used: ^a^AD + D versus. AD-D, ^b^AD-D versus. DLB + D.

^c^Postmortem delay indicates the number of hours between time of death and storage of the brain at −80°C; ^d^only MMSE scores with no more than four months between scoring and date of death were included.

AD + D/-D, depressed/nondepressed Alzheimer’s disease patients; Behave-AD, Behavioral Pathology in Alzheimer’s Disease Rating Scale; CMAI, Cohen-Mansfield Agitation Inventory; CONTR; control subjects; CSDD, Cornell Score for Depression in Dementia; DLB + D, depressed dementia with Lewy bodies patients; GDS, Global Deterioration Scale; MFS, Middelheim Frontality Score; MMSE, Mini-Mental State Examination; N/A, not applicable.

**Table 2:** **Focus depression: comparison of the brain monoamine levels between AD + D, AD-D, DLB + D and CONTR**

**Brain region**	**MA and MT or ratio**	**AD + D (number = 10)**	**AD-D (number = 10)**	**DLB + D (number = 10)**	**CONTR (number = 10)**
BA9	MHPG (ng/g)	412.4 (314.3-704.8); n = 9	1,092.3 (499.2-1,734.5); n = 10^b^	284.3 (155.8-547.0); n = 10^b^	478.7 (409.2-758.3); n = 10
	5-HT (ng/g)	9.8 (5.3-16.9); n = 9^a^	12.6 (7.7-14.4); n = 10^e, bbb^	3.4 (2.5-4.2); n = 10^a, bbb^	4.2 (1.3-11.0); n = 10^e^
BA10	MHPG (ng/g)	684.2 (312.1-1,080.1); n = 10	663.9 (432.5-940.3); n = 10^b^	297.7 (143.5-460.4); n = 10^b^	289.9 (177.3-498.1); n = 10
	5-HIAA (ng/g)	239.2 (156.9-367.3); n = 10^a^	178.5 (124.3-310.5); n = 10	82.1 (58.9-125.8); n = 10^a^	128.7 (85.8-157.0); n = 10
	HVA (ng/g)	116.6 (89.2-132.0); n = 10^a^	82.8 (63.2-98.0); n = 10	57.3 (41.9-66.3); n = 10^a^	102.6 (66.1-138.7); n = 10
	5-HT (ng/g)	16.0 (11.8-34.4); n = 10^aaa^	13.4 (12.7-15.4); n = 10^bbb^	4.3 (2.4-5.8); n = 10^aaa,^ ^bbb, f^	12.4 (8.6-18.3); n = 10^f^
BA24	5-HIAA (ng/g)	394.5 (300.9-565.8); n = 10^a^	378.2 (360.6-508.6); n = 10^b^	229.5 (194.6-335.2); n = 10^a, b^	301.2 (192.1-499.8); n = 10
	HVA (ng/g)	214.5 (180.5-263.2); n = 10^a^	202.1 (144.0-224.2); n = 10	131.21 (100.5-183.1); n = 10^a^	210.6 (179.8-263.6); n = 10
	5-HT (ng/g)	39.4 (32.0-54.1); n = 10^aa^	44.4 (34.7-52.9); n = 10^bb^	21.6 (8.4-23.5); n = 10^aa, bb^	42.6 (22.0-53.0); n = 10
amygdala	MHPG (ng/g)	1,100.5 (634.4-1,568.7); n = 9^a^	592.2 (209.1-1,050.7); n = 8	430.1 (137.3-644.2); n = 10^a^	347.9 (241.9-588.1); n = 10
	NA (ng/g)	67.4 (54.7-395.9); n = 8	76.2 (61.0-127.9); n = 8^b^	36.2 (26.6-48.9); n = 10^b^	60.2 (46.4-81.5); n = 10
hippocampus	5-HIAA (ng/g)	383.2 (260.5-689.9); n = 10	385.0 (348.7-954.9); n = 9^b^	271.9 (239.1-331.6); n = 10^b^	284.4 (242.9-349.3); n = 10
	DA (ng/g)	9.0 (5.2-120.8); n = 9^a^	5.9 (4.7-16.7); n = 9	3.8 (2.4-5.3); n = 10^a^	8.9 (6.5-23.7); n = 10
	HVA (ng/g)	224.5 (157.6-269.6); n = 10	206.5 (132.0-249.9); n = 9	133.0 (108.7-168.0); n = 10^f^	236.1 (193.3-333.1); n = 10^f^
	5-HIAA/5-HT	8.2 (5.5-9.6); n = 10^a^	6.2 (4.2-13.8); n = 9	3.8 (2.8-5.2); n = 10^a^	4.4 (3.4-5.9); n = 10
	DOPAC/DA	0.4 (0.1-1.7); n = 9^a^	1.5 (0.5-2.3); n = 9	3.0 (1.6-3.7); n = 10^a, ff^	0.7 (0.4-1.2); n = 10^ff^
thalamus	MHPG (ng/g)	1,273.7 (776.8-1,740.1); n = 10^aa^	686.2 (521.1-1,585.9); n = 10	248.5 (174.0-568.9); n = 10^aa^	494.4 (315.2-1,125.7); n = 10
	NA (ng/g)	140.5 (111.1-164.6); n = 8^a^	183.9 (146.7-261.6); n = 10^b^	47.0 (25.0-76.6); n = 10^a, b, ff^	162.9 (91.6-213.7); n = 10^ff^
	DA (ng/g)	14.0 (8.6-31.6); n = 10^aaa^	13.1 (9.3-28.5); n = 10^bb^	1.4 (1.0-5.3); n = 10^aaa, bb, fff^	19.7 (8.7-31.8); n = 10^fff^
	DOPAC/DA	1.0 (0.5-1.3); n = 10^a^	1.0 (0.5-1.6); n = 10^b^	3.7 (1.9-11.3); n = 10^a, b, ff^	0.9 (0.5-1.1); n = 10^ff^
	HVA/DA	31.0 (20.7-42.2); n = 10^a^	29.2 (14.8-70.0); n = 10^b^	225.5 (55.8-361.9); n = 10^a, b, f^	29.2 (23.1-44.3); n = 10^f^
BA11	5-HIAA (ng/g)	238.6 (165.2-464.6); n = 9	293.9 (176.1-424.6); n = 10^b^	83.9 (65.9-132.3); n = 10^b, f^	222.3 (161.4-353.6); n = 10^f^
	HVA (ng/g)	146.1 (92.7-165.2); n = 9^a^	132.9 (84.3-182.5); n = 10^b^	62.5 (43.4-83.7); n = 10^a, b, f^	118.9 (91.7-166.1); n = 10^f^
	5-HT (ng/g)	11.8 (6.8-20.2); n = 9	17.3 (10.5-36.7); n = 10^bb^	5.9 (3.7-8.2); n = 10^bb^	9.0 (5.6-14.5); n = 10
	MHPG/NA	81.7 (37.6-111.1); n = 6^a^	47.1 (21.3-64.3); n = 10	21.5 (10.2-31.5); n = 10^a^	42.7 (33.1-69.9); n = 10
BA22	MHPG (ng/g)	779.9 (546.3-1,177.4); n = 10^aa^	810.3 (308.6-1,492.7); n = 10	296.0 (171.6-521.2); n = 10^aa^	609.6 (419.2-1,591.4); n = 10
	5-HIAA (ng/g)	472.3 (293.2-793.5); n = 10^aaa^	311.3 (166.6-958.3); n = 10^b^	64.4 (46.5-133.3); n = 10^aaa, b, f^	255.2 (127.0-473.0); n = 10^f^
	HVA (ng/g)	138.4 (125.5-207.8); n = 10^a^	116.8 (91.4-148.4); n = 10^b^	61.0 (47.1-89.0); n = 10^a, b, f^	141.7 (86.0-240.0); n = 10^f^
	MHPG/NA	83.5 (23.6-92.1); n = 7	46.5 (29.1-137.8); n = 9^b^	17.9 (9.0-29.5); n = 10^b^	33.9 (22.1-79.3); n = 10
BA17	5-HIAA (ng/g)	143.6 (98.1-191.0); n = 9^aa^	117.8 (92.1-187.9); n = 10^b^	47.8 (32.6-69.5); n = 10^aa, b, ff^	147.9 (118.4-198.0); n = 10^ff^
	DA (ng/g)	12.2 (7.1-17.9); n = 9^d,^ ^c^	2.7 (1.5-3.8); n = 10 ^c^	4.3 (2.6-7.1); n = 10	3.5 (1.6-4.8); n = 10^d^
	5-HT (ng/g)	13.7 (6.5-24.1); n = 9^a^	12.7 (3.4-24.4); n = 10	2.6 (2.2-5.7); n = 10^a, f^	8.8 (5.5-20.3); n = 10^f^
Cerebellum	5-HIAA (ng/g)	244.3 (103.4-538.2); n = 9^a^	276.3 (117.6-465.3); n = 9^b^	57.3 (38.4-81.8); n = 10^a, b, f^	181.9 (103.3-333.6); n = 10^f^
	DA (ng/g)	5.6 (2.4-7.1); n = 9^a^	3.0 (2.0-17.9); n = 9	2.3 (0.5-2.6); n = 10^a^	3.4 (2.2-4.7); n = 10
	HVA (ng/g)	105.4 (51.1-137.6); n = 9^a^	64.5 (55.9-94.9); n = 9^b^	35.3 (22.9-59.5); n = 10^a, b^	94.5 (43.1-149.2); n = 10
	DOPAC/DA	2.3 (1.0-5.8); n = 9	1.7 (0.3-3.9); n = 9^b^	6.7 (3.7-13.9); n = 10^b^	1.4 (0.7-6.0); n = 9
Locus coeruleus	DOPAC (ng/g)	55.5 (32.8-102.6); n = 9^a^	41.5 (28.1-101.6); n = 9	19.3 (12.7-28.1); n = 10^a, ff^	78.8 (47.1-130.3); n = 10^ff^
	DA (ng/g)	41.0 (25.9-102.9); n = 9^a^	34.5 (20.0-49.4); n = 9	15.8 (7.0-25.0); n = 10^a, f^	37.1 (30.1-55.7); n = 10^f^
	HVA (ng/g)	1,361.6 (963.2-1,904.9); n = 9^a^	1,082.7 (834.7-1,634.6); n = 9^b^	540.2 (378.5-887.8); n = 10^a, b, fff^	1,603.2 (1,284.7-1,964.1); n = 10^fff^
	HVA/5-HIAA	0.4 (0.2-0.5); n = 9	0.2 (0.2-0.3); n = 9	0.2 (0.1-0.2); n = 10^f^	0.4 (0.3-0.5); n = 10^f^
	MHPG/NA	1.1 (0.9-2.6); n = 9	1.5 (1.3-2.0); n = 9^b^	3.9 (2.1-4.8); n = 10^b^	0.8 (0.5-2.4); n = 10

Median (IQR); Kruskal-Wallis analyses (*P* <0.05) with *post-hoc* Mann–Whitney U tests were performed; only data remaining statistically significant following Bonferroni correction for multiple comparisons (*P* <0.00833; one superscript letter) are presented above; significant differences with *P* <0.001 and *P* <0.0001 are respectively indicated with two and three repeated superscript letters; the following letters are used: ^a^AD + D vs. DLB + D, ^b^AD-D vs. DLB + D, ^c^AD + D vs. AD-D, ^d^AD + D vs. CONTR, ^e^AD-D vs. CONTR and ^f^DLB + D vs. CONTR.

5-HIAA, 5-hydroxyindoleacetic acid; 5-HT, 5-hydroxytryptamine (serotonin); AD + D/-D, depressed/nondepressed Alzheimer’s disease patients; BA, Brodmann area; CONTR, control subjects; DA, dopamine; DLB + D, depressed dementia with Lewy bodies patients; DOPAC, 3,4-dihydroxyphenylacetic acid; HVA, homovanillic acid; MA and MT, monoamines and metabolites; MHPG, 3-methoxy-4-hydroxyphenylglycol; NA, noradrenaline.

**Table 3:** **Focus psychosis: comparison of the brain monoamine levels between AD + D + P, AD + D-P, DLB + D + P and DLB + D-P**

**Brain region**	**MA and MT or ratio**	**AD + D + P (number = 5)**	**AD + D-P (number = 5)**	**DLB + D + P (number = 5)**	**DLB + D-P (number = 5)**
BA9	HVA (ng/g)	146.6 (85.1-198.0); n = 4	127.2 (117.8-152.5); n = 5^b^	110.7 (81.8-156.2); n = 5	51.5 (44.1-73.0); n = 5^b^
	5-HT (ng/g)	5.3 (3.3-15.8); n = 4	14.1 (8.7-76.8); n = 5^b, d^	4.2 (1.7-5.2); n = 5^d^	3.4 (2.6-3.5); n = 5^b^
BA10	5-HIAA (ng/g)	227.6 (99.6-331.1); n = 5	250.9 (169.7-441.7); n = 5^d^	70.0 (56.1-128.9); n = 5^d^	87.7 (63.2-180.0); n = 5
	HVA (ng/g)	111.3 (84.7-131.7); n = 5^c^	117.9 (95.0-139.2); n = 5^b^	62.1 (59.2-104.8); n = 5	45.0 (31.9-54.4); *n* = 5^c, b^
	5-HT (ng/g)	16.0 (10.4-35.8); n = 5^a, c^	16.1 (10.8-161.0); n = 5^b, d^	4.6 (1.6-5.6); n = 5^a, d^	4.1 (2.9-6.0); n = 5^c, b^
BA24	HVA (ng/g)	212.8 (163.4-265.0); *n* = 5	216.2 (185.3-263.1); n = 5^b^	167.7 (131.2-206.1); n = 5	102.9 (79.6-140.2); n = 5^b^
	5-HT (ng/g)	32.2 (29.3-48.7); n = 5	47.0 (39.4-165.6); n = 5^d^	18.9 (7.1-24.6); n = 5^d^	22.0 (14.9-32.0); n = 5
Hippocampus	MHPG (ng/g)	459.5 (224.9-1,000.7); n = 5	1,078.1 (850.3-1,099.3); n = 5^d^	358.9 (171.2-569.5); n = 5^d^	383.1 (318.3-570.7); n = 5
	HVA (ng/g)	193.9 (146.5-462.3); n = 5	245.6 (174.4-284.7); n = 5^b^	143.3 (114.4-250.4); n = 5	116.9 (106.4-138.6); n = 5^b^
Thalamus	MHPG (ng/g)	793.0 (678.3-1,344.8); n = 5	1,342.5 (1,273.7-1,860.6); n = 5^b, d^	245.5 (143.1-569.9); n = 5^d^	251.6 (164.9-697.1); n = 5^b^
	DA (ng/g)	17.0 (7.1-22.7); n = 5	11.1 (9.3-857.5); n = 5^b^	1.1 (1.0-5.3); n = 5	1.7 (0.8-5.6); n = 5^b^
	HVA/DA	32.0 (25.5-53.1); n = 5	30.1 (5.7-42.4); n = 5^d^	230.5 (146.2-382.8); n = 5^d^	137.7 (26.5-439.3); n = 5
BA22	5-HIAA (ng/g)	756.7 (241.2-1,147.4); n = 5	450.2 (332.4-507.9); n = 5^b^	64.0 (44.1-162.6); n = 5	81.3 (49.0-158.8); n = 5^b^
	HVA (ng/g)	181.7 (135.6-218.4); n = 5^a, c^	129.5 (80.6-178.2); n = 5	72.0 (61.0-104.0); n = 5^a^	50.8 (31.3-69.5); n = 5^c^
BA17	5-HIAA (ng/g)	138.3 (93.9-189.9); n = 4	143.6 (98.1-215.2); n = 5^d^	43.1 (22.4-60.3); n = 5^d^	54.7 (38.4-110.8); n = 5
	5-HT (ng/g)	9.8 (4.2-19.5); n = 4	14.5 (7.4-26.8); n = 5^b^	2.5 (1.6-5.2); n = 5	3.5 (2.5-5.9); n = 5^b^
Locus coeruleus	DA (ng/g)	32.3 (20.2-132.4); n = 5^c^	52.5 (33.0-102.5); n = 4	21.1 (12.0-42.1); n = 5	12.1 (6.5-16.5); n = 5^c^
	HVA (ng/g)	1,361.6 (963.2-1,908.4); n = 5^c^	1,225.4 (867.6-1,834.0); n = 4	884.8 (506.1-1,013.5); n = 5	408.0 (319.8-648.7); n = 5^c^
	HVA/5-HIAA	0.3 (0.2-0.6); n = 5^c^	0.4 (0.2-0.5); n = 4	0.2 (0.2-0.4); n = 5	0.1 (0.08-0.2); n = 5^c^

Median (IQR); Kruskal-Wallis analyses (*P* <0.05) with *post-hoc* Mann-Whitney U tests were performed; only data remaining statistically significant following Bonferroni correction for multiple comparisons (*P* <0.00833; one superscript letter) are presented above; the following letters are used: ^a^AD + D + P vs. DLB + D + P, ^b^AD + D-P vs. DLB + D-P, ^c^AD + D + P vs. DLB + D-P and ^d^AD + D-P vs. DLB + D + P.

5-HIAA, 5-hydroxyindoleacetic acid; 5-HT, 5-hydroxytryptamine (serotonin); AD + D + P/-P, psychotic/nonpsychotic Alzheimer’s disease patients (within the depressed AD group); BA, Brodmann area; DA, dopamine; DLB + D + P/-P, psychotic/nonpsychotic dementia with Lewy bodies patients (within the depressed DLB group); HVA, homovanillic acid; MA and MT, monoamines and metabolites; MHPG, 3-methoxy-4-hydroxyphenylglycol.

**Table 4:** **Significant brain monoaminergic correlates of NPS in DLB and AD**

**Study group**	**Brain region**	**MA and MT or ratio**	**NPS feature**	**Spearman’s Rank Order correlation statistics**
**DLB patients**				
Number = 10				
	BA10	DA (ng/g tissue)	CMAI cluster 3	*R* = +0.841, *P* = 0.002, number = 10
			verbal agitation	
	hippocampus	DA (ng/g tissue)	Behave-AD cluster AB	***R*** **= +0.928,** ***P*** **= 0.0001, **number **= 10**
			psychosis	
		DA (ng/g tissue)	Behave-AD total score	*R* = +0.825, *P* = 0.003, number = 10
	thalamus	DA (ng/g tissue)	CSDD total scores	*R* = +0.839, *P* = 0.002, number = 10
			depression	
		HVA/DA ratio	CSDD total scores	*R* = −0.851, *P* = 0.002, number = 10
			depression	
	BA22	DA (ng/g tissue)	CMAI total score	*R* = +0.827, *P* = 0.003, number = 10
			agitation	
	cerebellum	DOPAC/DA ratio	Behave-AD cluster F	*R* = −0.846, *P* = 0.002, number = 10
			affective disturbances	
**AD patients**				
Number = 20				
	amygdala	DA (ng/g tissue)	Behave-AD cluster AB	***R*** **= −0.728,** ***P*** **= 0.0009, number = 17**
			psychosis	
		HVA/DA ratio	Behave-AD cluster AB	***R*** **= +0.766,** ***P*** **= 0.0003, number = 17**
			psychosis	
		HVA/DA ratio	CMAI cluster 3	***R*** **= +0.786,** ***P*** **= 0.0002, number = 17**
			verbal agitation	
		HVA/DA ratio	CMAI total score	*R* = +0.671, *P* = 0.003, number = 17
			agitation	
	hippocampus	HVA/5-HIAA ratio	Behave-AD cluster C	*R* = +0.659, *P* = 0.002, number = 19
			activity disturbances	
	BA17	HVA/DA ratio	Behave-AD cluster F	*R* = −0.670, *P* = 0.002, number = 19
			affective disturbances	
		DA (ng/g tissue)	CSDD total scores	*R* = +0.662, *P* = 0.002, number = 19
			depression	
	LC	MHPG/NA ratio	Behave-AD cluster D	*R* = +0.727, *P* = 0.001, number = 16
			agitation and aggression	

Significant correlations between NPS and neurochemical data in postmortem brain tissue samples of DLB and AD patients as indicated by Spearman’s Rank Order correlation statistics (correlation coefficient (*R*), *P* value and sample size (n)). Only correlations remaining statistically significant following Bonferroni correction are displayed above (*P* <0.0033). The four most significant correlations (*P* <0.001) are shown in **bold**. 5-HIAA, 5-hydroxy-3-indoleacetic acid; AD, Alzheimer’s disease; BA, Brodmann area; Behave-AD, Behavioral Pathology in Alzheimer’s Disease Rating Scale; CMAI, Cohen-Mansfield Agitation Inventory; CSDD, Cornell Scale for Depression in Dementia; DA, dopamine; DLB, dementia with Lewy bodies; DOPAC, 3,4-dihydroxyphenylacetic acid; HVA, homovanillic acid; LC, locus coeruleus; MA and MT, monoamines and metabolites; MFS, Middelheim Frontality Score; MHPG, 3-methoxy-4-hydroxyphenylglycol; NA, noradrenaline; NPS, neuropsychiatric symptoms.

## Results

### Clinical and MMSE data, pH values and dementia staging

Table 1 summarizes the clinical, MMSE, pH and GDS data.

All groups were age- and gender-matched, with similar storage times of the analyzed brain tissue samples and postmortem delays. The number of patients who were on psychotropic medication before death compared to the patients who were free of such medication was comparable between groups as well. The different types of administered psychotropic medication were cholinomimetics (n = 1 for AD + D; n = 2 for AD-D; n = 3 for DLB + D), prolopa/levodopa (n = 3 for DLB + D), antidepressants (n = 4 for AD + D; n = 1 for AD-D; n = 3 for DLB + D; n = 2 for controls), hypnotics/sedatives/anxiolytics (n = 1 for AD + D and DLB + D; n = 2 for AD-D; n = 3 for controls) and antipsychotics (n = 4 for AD + D and AD-D; n = 2 for DLB + D). The average number of days between the last behavioral assessment and date of death was 3.8, 3.9 and 5.2 days for the AD + D, AD-D and DLB + D groups, respectively. Furthermore, MMSE scores of the DLB + D group were significantly lower than those of the AD-D group (*P* = 0.013), who had the overall highest MMSE scores. Finally, GDS scores and pH values were comparable between groups with, in total, one AD + D and one AD-D patient who had low cerebellar pH values (<6.1). For these patients, extra pH analyses on the ten remaining brain regions were performed. Eventually, brain samples with acidotic pH values were excluded from statistical analysis, that is, for BA9 (n = 1), BA11 (n = 1), BA17 (n = 1), amygdala (n = 1) and cerebellum (n = 2).

### Behavioral assessment scores

The behavioral assessment scores of each group are presented in Table 1.

The MFS total scores (*P* = 0.004), Behave-AD total scores (*P* = 0.005), Behave-AD global scores (*P* = 0.004), CMAI cluster 2 scores (*P* = 0.015) and CSDD total scores (*P* = 0.00001) were significantly higher in the AD + D group compared to the AD-D group, whereas the Behave-AD cluster B scores (*P* = 0.013), Behave-AD cluster AB scores (*P* = 0.013) and CSDD total scores (*P* = 0.00001) were all significantly lower in the AD-D group compared to the DLB + D group.

### Focus depression: neurochemical comparison of brain monoamines, metabolites and ratios between AD + D, AD-D, DLB + D and control subjects

Only the neurochemical group differences which remained statistically significant following Bonferroni correction are displayed in Table 2 (*P* <0.00833). Nonsignificant data were omitted.

The DLB + D group had the overall lowest monoamine and metabolite levels, as well as ratios. More specifically, the DLB + D group had the significantly lowest values for 33 out of 41 monoaminergic group alterations (rows), across 11 brain regions, compared to AD + D, AD-D and/or control subjects (Table 2). In addition, the DLB + D group had the significantly highest DOPAC/DA and HVA/DA ratios, indicative of dopaminergic turnover, specifically for hippocampus (only DOPAC/DA), thalamus (both), and cerebellum (only DOPAC/DA), as well as the highest MHPG/NA ratios, indicative of noradrenergic catabolism, in the LC. The control group had the lowest, second lowest and highest values for 5, 18 and 7 out of 41 significant monoaminergic group differences, respectively. As for the AD + D/-D groups, levels varied, but, in general, they had the highest concentrations for 29 rows out of the total of 41.

The most statistically significant alterations were noticeable in the thalamus, with significantly lower DA levels and, at the same time, significantly increased DOPAC/DA and HVA/DA ratios in the DLB + D population compared to the AD + D (*P* = 0.00008, 0.002 and 0.007, respectively), AD-D (*P* = 0.0003, 0.002 and 0.007, respectively) and control group (*P* = 0.00008, 0.0003 and 0.004, respectively). Thalamic NA levels were also significantly decreased (*P* = 0.003, 0.001 and 0.0005, respectively), whereas thalamic MHPG levels of the DLB + D group were only significantly lower when compared to the AD + D population (*P* = 0.0002). Similarly, 5-HT levels of BA9, BA10 and BA24, as well as 5-HIAA levels of BA10, BA17, BA22 and BA24 were significantly lower in DLB + D patients compared to their AD + D (for 5-HT: *P* = 0.001, 0.00001 and 0.0005; for 5-HIAA: *P* = 0.003, 0.00008, 0.0004 and 0.007, respectively) and AD-D (for 5-HT: *P* = 0.00008, 0.00008 and 0.0003; for 5-HIAA: *P* = 0.029, 0.002, 0.001 and 0.007, respectively) counterparts. The hippocampal 5-HIAA/5-HT ratios, indicative of serotonergic catabolism, were significantly decreased in DLB + D patients compared to AD + D patients as well (*P* = 0.003). Furthermore, in the LC, DOPAC, DA and HVA levels of the DLB + D population were significantly decreased compared to the AD + D (*P =* 0.003, 0.003 and 0.001, respectively) and control group (*P =* 0.0001, 0.002 and 0.00001, respectively) (Table 2).

Finally, DA levels of BA17 were significantly increased in AD + D patients compared to AD-D patients and the control group (*P* = 0.003 and 0.006, respectively), whereas 5-HT levels of BA9 were significantly higher in AD-D patients compared to the control subjects (*P* = 0.007).

### MHPG levels across different postmortem brain regions in DLB versus AD and controls

**Figure 1:**
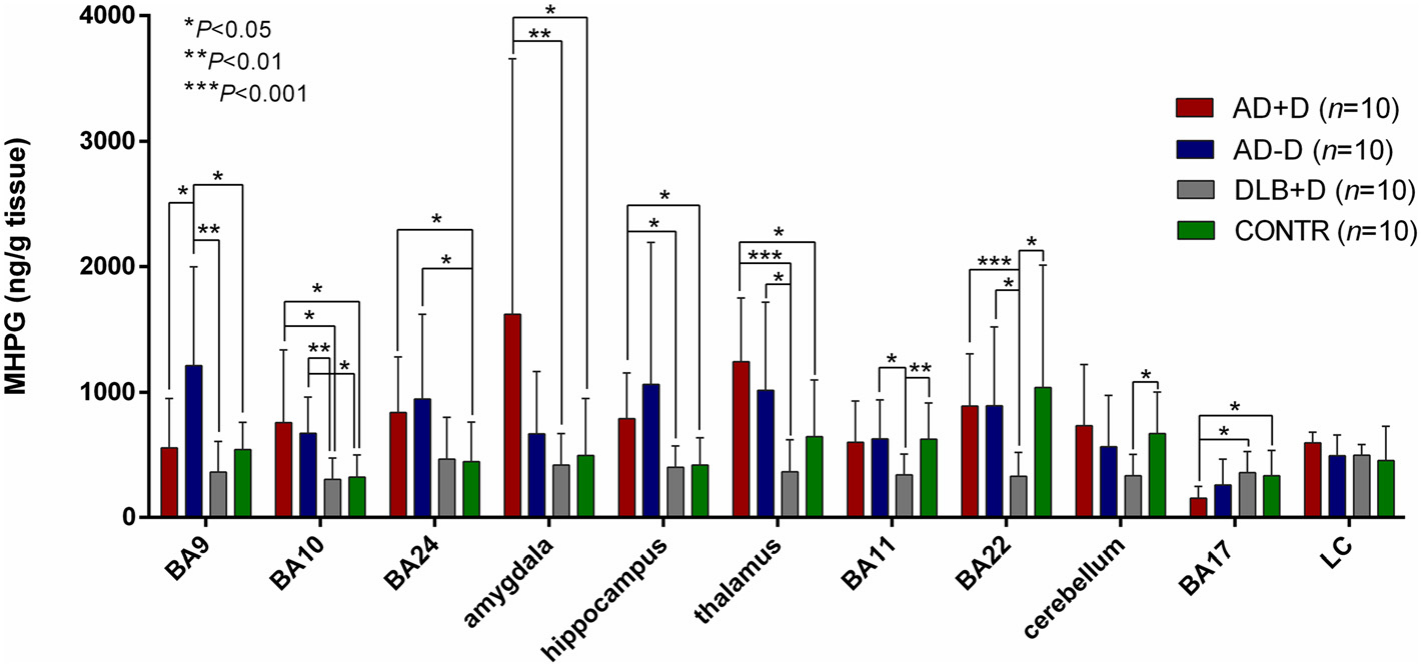
**MHPG concentrations across different brain regions in DLB** + **D compared to AD** + **D, AD-D and CONTR.** Data are presented as mean with SD. Nonparametric Mann–Whitney U statistics were performed. MHPG levels of seven out of eleven postmortem brain regions were significantly decreased in DLB + D compared to AD + D and/or AD-D patients (*P* values vary from <0.05 to <0.001). In BA17, MHPG concentrations of DLB + D patients were significantly higher. AD + D/-D, depressed/nondepressed Alzheimer’s disease patients; BA, Brodmann area; CONTR, control subjects; DLB + D, depressed dementia with Lewy bodies patients; LC, locus coeruleus; MHPG, 3-methoxy-4-hydroxyphenylglycol.

Remarkably, MHPG was significantly decreased in seven out of eleven postmortem brain regions of DLB subjects compared to AD + D and/or AD-D patients, that is, BA9 (*P* = 0.004 compared to AD-D), BA10 (*P* = 0.04 and 0.008 compared to AD + D and AD-D, respectively), amygdala (*P* = 0.007 compared to AD + D), hippocampus (*P* = 0.023 compared to AD + D), thalamus (*P* = 0.0002 and 0.013 compared to AD + D and AD-D, respectively), BA11 (*P* = 0.028 compared to AD-D) and BA22 (*P* = 0.0004 and 0.041 compared to AD + D and AD-D, respectively) (Figure 1). As for BA24, MHPG levels were almost significantly lower in DLB patients compared to both AD + D and AD-D subjects (borderline significance; *P* = 0.059 for both). Surprisingly, in BA17, DLB patients as well as control subjects had significantly higher MHPG levels compared to their AD + D counterparts (*P* = 0.011 for both). In total, for five out of seven brain regions in which statistically different MHPG levels were found (*P* <0.05), these differences remained significant following Bonferoni correction for multiple comparisons (Table 2; Figure 1).

For the cerebellar cortex and the LC, no MHPG differences were spotted between the DLB and AD groups, except for NA levels and MHPG/NA ratios in the LC of DLB patients, which were respectively significantly decreased and increased compared to both AD + D and AD-D patients (for AD + D: *P* = 0.041 for both; for AD-D: *P* = 0.018 and 0.007 (Table 2)).

Besides the DLB-AD comparison, MHPG concentrations were significantly increased in the AD + D and/or AD-D group(s) compared to the control group as well, that is, for BA9 (*P* = 0.049 compared to AD-D), BA10 (*P* = 0.04 and 0.01 compared to AD + D and AD-D, respectively), BA24 (*P* = 0.03 and 0.049 compared to AD + D and AD-D, respectively), amygdala (*P* = 0.027 compared to AD + D), hippocampus (*P* = 0.027 compared to AD + D) and thalamus (*P* = 0.016 compared to AD + D). As for BA17, MHPG levels of the control group were significantly increased compared to the AD + D group (*P* = 0.011). Finally, in BA11, BA22 and cerebellar cortex, we also noticed significantly lower MHPG levels in DLB patients compared to control subjects (*P* = 0.007, 0.011 and 0.023, respectively) (Figure 1).

### Focus psychosis: neurochemical comparison of brain monoamines, metabolites and ratios between AD + D + P, AD + D-P, DLB + D + P and DLB + D-P

Table 3 summarizes the brain monoaminergic differences between the subgroups of psychotic and nonpsychotic AD and DLB patients that remained statistically significant following Bonferroni correction for multiple comparisons (*P* <0.00833). Nonsignificant data were excluded from the Table.

With regard to the DLB + D + P and AD + D + P subgroups, statistical analyses showed that 5-HT levels of BA10, as well as HVA levels of BA22 were significantly lower in DLB + D + P patients compared to AD + D + P patients (*P* = 0.008 for both). Similarly, 5-HT levels of BA9, BA10 and BA17, as well as 5-HIAA levels of BA22 and HVA levels of BA9, BA10, BA24 and the hippocampus, were all significantly lower in DLB + D-P patients compared to AD + D-P patients (*P* = 0.008 for all; Table 3).

Notably, in the amygdala, DOPAC and DA levels, as well as HVA/DA ratios, were significantly decreased and increased, respectively, in AD + D + P patients compared to AD + D-P patients, although significance was not maintained following a Bonferroni correction for multiple comparisons (data not shown; for DOPAC and DA: *P* = 0.032 and 0.014; for HVA/DA: *P* = 0.016). The same applies for the monoaminergic group comparison of DLB + D + P/-P, with significantly increased HVA levels in BA9, BA10, BA11, BA22 and BA24, as well as increased DA levels in the hippocampus and increased HVA/5-HIAA ratios in the LC, indicating the inhibitory effect of the serotonergic system on dopaminergic neurotransmission [62,63] of DLB + D + P patients compared to DLB + D-P patients (data not shown; for HVA_BA9/BA10/BA11_ and HVA/5-HIAA_LC_: *P* = 0.016; for HVA_BA22/BA24_: *P* = 0.047; for DA_hippocampus_: *P* = 0.032).

### Brain monoaminergic correlates of NPS features in the DLB and AD population

Table 4 shows the significant brain monoaminergic correlates of NPS in the DLB and total AD group, only for those that remained significant following Bonferroni correction. Both AD subgroups were joined into one total AD group (n = 20) in order to obtain a heterogeneous cohort with a better distribution of NPS scores since AD + D patients had much higher behavioral scores compared to AD-D patients (Table 1). Furthermore, there were no significant brain monoamine differences between AD + D and AD-D subjects, except of DA levels in BA17 (Table 2). The merging also resulted in increased statistical power. The four most significant correlations are described below.

**Figure 2:**
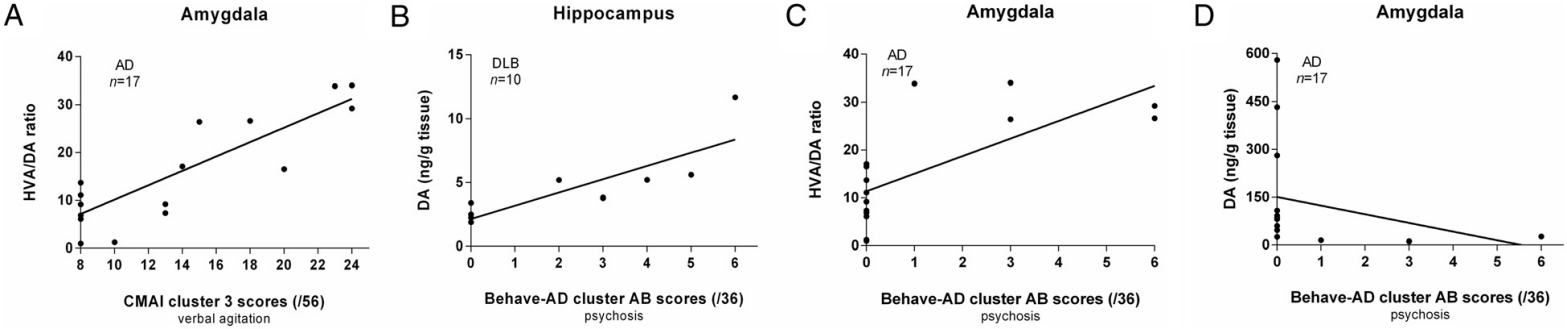
**Scatter plots representing the four most significant NPS correlates of altered brain monoamine levels in AD and DLB. A**. *R* = +0.786, *P* = 0.0002; **B**. *R* = +0.928, *P* = 0.0001; **C**. *R* = +0.766, *P* = 0.0003; **D**. *R* = −0.728, *P* = 0.0009. AD, Alzheimer’s disease; Behave-AD, Behavioral Pathology in Alzheimer’s Disease Rating Scale; CMAI, Cohen-Mansfield Agitation Inventory; DA, dopamine; DLB, dementia with Lewy bodies; HVA, homovanillic acid; NPS, neuropsychiatric symptoms.

In the DLB group, hippocampal DA levels strongly correlated with Behave-AD cluster AB scores (psychosis) (*P* = 0.0001, *R* = +0.928, n = 10; Figure 2B).

In the total AD population, HVA/DA ratios of the amygdala strongly correlated with Behave-AD cluster AB scores (psychosis) (*P* = 0.0003, *R* = +0.766, n = 17; Figure 2C) and CMAI cluster 3 scores (verbal agitation) (*P* = 0.0002, *R* = +0.786, n = 17; Figure 2A), whereas DA levels of the amygdala inversely correlated with Behave-AD cluster AB scores (psychosis) (*P* = 0.0009, *R* = −0.728, n = 17; Figure 2D). Noteworthy, MHPG/NA ratios of the LC significantly correlated with Behave-AD cluster D scores (agitation/aggression) in the total AD group as well (*P* = 0.001, *R* = +0.728, n = 16), although statistical significance was not maintained after Bonferroni correction.

### Potential confounding monoaminergic effects of administered psychotropic medication

In the AD + D group, HVA levels of the amygdala (727.8 versus 537.0 ng/ml; *P* = 0.016), MHPG levels of BA17 (164.6 versus 119.6 ng/ml; *P* = 0.016) and MHPG (669.9 versus 531.4 ng/ml; *P* = 0.029) and HVA levels of the LC (1,690.0 versus 963.2 ng/ml; *P* = 0.029) were significantly higher in patients who were administered psychotropic medication before death (n = 4), compared to patients free of such medication (n = 5). In AD-D patients, only HVA/5-HIAA ratios of BA17 were revealed to be significantly lower in those patients who took psychotropic medication (n = 7) compared to their medication-free counterparts (n = 3) (0.259 versus 0.541; *P* = 0.017). With regard to the control subjects, only MHPG levels of BA22 were significantly increased in takers (n = 5) versus non-takers (n = 5) (1,484.0 versus 428.7 ng/ml; *P* = 0.032) and finally, in the DLB + D group, 5-HT levels of BA17 were significantly lower (2.443 versus 4.822 ng/ml; *P* = 0.038), whereas HVA levels of the cerebellar cortex (51.7 versus 20.8 ng/ml; *P* = 0.019) as well as the HVA/5-HIAA ratios of BA9 (1.408 versus 0.459; *P* = 0.038), BA11 (0.839 versus 0.378; *P* = 0.01) and BA24 (0.685 versus 0.325; *P* = 0.01) were all significantly higher in patients on psychotropic medication (n = 6) compared to patients free of such drugs (n = 4).

## Discussion

### Study strengths and weaknesses

Following our study protocol which allows no more than six hours between death and brain storage at −80°C, an average postmortem delay of approximately three to four hours was accomplished for each group. Besides this short time interval, additional pH measurements as a second quality control measure also ensured us of non-acidotic and high quality brain tissue samples. All groups were age- and gender-matched, with similar storage times of the frozen brain tissue samples and a low number of days between the last behavioral assessment and date of death (Table 1; see Results). Moreover, all the paraformaldehyde-fixated right brain hemispheres were always neuropathologically examined by the same neuropathologist and, in addition, regional brain dissections of the left frozen hemispheres were also carried out by the same scientist, which, respectively, minimized the diagnostic and inter-dissection variability. Our optimized RP-HPLC-ECD method was reliable, fast, robust and sensitive for the simultaneous determination of eight monoamines and metabolites with a low detection limit, high repeatability and recovery, short runtime, measurements in duplicate, short and simple sample preparation procedure and relatively high throughput. All analyses and sample preparation procedures were accurately performed by the same two scientists, which resulted in a low inter-assay variability.

With regard to the study weaknesses, each group consisted of a relatively small number of patients, although each patient was clinically, behaviorally and neuropathologically well characterized and nonparametric statistics were applied. Unfortunately, no behavioral ratings were available for the control subjects, although their clinical records did not demonstrate any evidence of psychiatric antecedents or depression. The absence of a non-depressed DLB group for neurochemical and statistical group comparisons might also be regarded as a study weakness. Furthermore, the administration of psychotropic medication before death might have camouflaged certain NPS and might also have altered the brain’s monoamine levels and -receptors. Our neurochemical data thus might have been influenced, since the neurochemical effects may last up to several weeks after the last administration. In order to address this weakness, we applied a total Bonferroni correction to all statistical analyses and compared the brain monoamine levels between patients taking and not taking psychotropic medication in each group. Accordingly, these analyses showed that all of the highly significant aforementioned brain monoamine differences between the groups were most likely not influenced by psychotropic drugs, except HVA levels in the LC of AD + D patients and 5-HT and HVA levels in BA17 and the cerebellum, respectively, of DLB + D patients. These specific monoamines were significantly altered in AD + D and DLB + D patients who were on psychotropic medication (see Results), which might have caused these same monoamines to become significantly altered in the AD + D/DLB + D comparison (Table 2). Lastly, the concurrent AD pathology in eight of ten neuropathologically confirmed DLB patients might also form a limitation of this study, although the co-occurrence of AD and DLB is very common. More specifically, the neuropathological overlap between both neurodegenerative conditions is so extensive that ‘pure’ DLB, that is, without any AD-related pathology beyond that attributable to normal aging, is relatively uncommon, accounting for merely one third of all cases of Lewy body dementia and perhaps 4% of all dementia cases [64].

### The serotonergic neuropathophysiology of depression in DLB compared to AD

Our results indicate that the serotonergic neurotransmission in the prefrontal, temporal, limbic and even occipital cortex as well as hippocampus might be severely impaired in DLB patients who suffered from depression. More specifically, we observed the lowest 5-HT and 5-HIAA levels as well as 5-HIAA/5-HT turnover ratios in eight of eleven brain regions of DLB + D patients compared to AD + D patients.

In general, LB accumulate in the dorsal raphe nucleus, which is the largest serotonergic nucleus in the human brain and provides a substantial proportion of the serotonergic innervation to the forebrain [65], resulting in a marked reduction of 5-HT levels in the striatum, neocortex and frontal cortex of DLB patients [25]. Ballard and colleagues [3] also adduced that the pathophysiology of depression in DLB, as well as PDD, is fundamentally different from that seen in AD, with alterations within the serotonergic pathway as a common feature of both. A previous study that investigated the theory of an impaired serotonergic neurotransmission in DLB patients with a major depressive disorder, observed an increased expression of 5-HT transporter (5-HTT) reuptake sites in BA7, the parietal neocortex, compared to those without depression [66]. On the other hand, in depressed AD patients, 5-HTT reuptake sites of the temporal cortex were reported to be significantly reduced compared to their nondepressed counterparts [67]. The depressed and nondepressed DLB groups of Ballard *et al*. [66] apparently also had lower mean 5-HTT reuptake site binding values than controls [unpublished observations; [25]]. The latter is comparable with our own serotonergic data, with significantly lower 5-HT and/or 5-HIAA levels in BA10, BA11, BA22, BA17 and cerebellum of DLB + D patients compared to healthy controls. In other studies of Sharp *et al*., higher 5-HT_1A_ receptor binding densities were examined in postmortem brain tissue samples of the frontal cortex and BA36 in DLB patients compared to healthy controls (for the frontal cortex) [52] and in a subgroup of DLB + D subjects compared to DLB-D patients (for BA36) [28].

Based upon these recent findings, it seems plausible that in response to the severely damaged and degenerated raphe nuclei, a selective postsynaptic 5-HT_1A_ receptor upregulation and relative preservation of the 5-HTT reuptake sites might be triggered, specifically in depressed DLB patients, to compensate for the decreased serotonergic neurotransmission from these nuclei towards their neocortical and limbic projection areas [3,25].

### Thalamic dopaminergic dysfunctioning and depression in DLB compared to AD

Besides the serotonergic brain differences, our monoaminergic data also point to a decreased dopaminergic neurotransmission with four- and seven-fold increases of DOPAC/DA and HVA/DA turnover ratios and a ten-fold decrease of DA levels in the thalamus of DLB + D compared to AD + D patients and control subjects. These findings in the thalamus might be attributed to the loss of DA producing neurons in the SN, one of the neuropathological hallmarks in DLB, although a significant reduction of nigro-thalamic and -striatal DA levels may also mediate some of the non-motor symptoms [29], such as depression. With regard to apathy, for example, David and colleagues [30] studied the relationship between apathy and striatal DA transporter (DAT) uptake in AD (n = 14) and DLB (n = 8) subjects by means of ^123^I-FP-CIT-SPECT imaging. The authors concluded that, using the Apathy Inventory, lack of initiative inversely correlated with bilateral putamen DAT uptake in the overall population of 22 patients. Although no statistical comparative analysis of the DAT binding potential (BP) values between DLB and AD was performed, their data clearly indicated that the subgroup of DLB subjects with apathy (n = 6; scores of 9 to 12) had much lower DAT BP values in left and right putamen than their apathetic AD counterparts (n = 3; scores of 9 to 12). On the other hand, increased thalamic D2 receptor densities in 18 DLB patients compared to 14 elderly controls have been reported as well [29], although the authors did not determine thalamic DA levels nor classify their DLB population into depressed/nondepressed study patients.

Interestingly, Wilson *et al*. [68] also noticed a strong association between the LB density in the LC, dorsal raphe nucleus as well as SN, and the degree of depressive symptoms in 124 older nondemented individuals. On the contrary, neurofibrillary tangle density within the same nuclei was not significantly related to depressive symptoms. Again, these results underline the prominent place of depression in synucleinopathies, such as DLB, and evidence the putative link between depression, LB and altered serotonergic and dopaminergic neurotransmitter systems.

### Psychosis and its dopaminergic pathogenesis in DLB versus AD

Based upon our findings, we suggest that psychosis in AD might be pathophysiologically associated with a decreased dopaminergic neurotransmission solely restricted to the amygdala, whereas an impaired and rather increased dopaminergic activity across the mesolimbic system and LC might clinically account for psychosis in DLB.

The pathogenic link between altered dopaminergic pathways and psychosis in DLB has not been intensively investigated before. The most intriguing evidence comes from Roselli *et al*. [31], who observed an association between decreased striatal DAT levels, measured by ^123^I-FP-CIT-SPECT imaging, and the presence of visual hallucinations in 18 DLB patients. When the authors considered the putamen and caudate nucleus separately, delusions, apathy and depression were inversely correlated with decreased caudate DAT levels also. The ratios of specific to nonspecific binding of ^123^I-FP-CIT, a DAT-specific radiotracer, were calculated as size-weighted averages of right, as well as left, brain activities. Similarly, our research group previously examined an inverse correlation between CSF HVA levels and hallucinations in a group of 26 DLB patients [33]. Overall, given the significantly impaired serotonergic neurotransmission in DLB brain [25], it is conceivable that the functional coupling between the serotonergic and dopaminergic pathways, with an inhibitory output of the serotonergic on the dopaminergic neurotransmission [62,63], could be severely impaired, possibly leading to increased HVA and DA levels. Such a disruption of a functionally coupled system might explain the overall serotonergic and dopaminergic differences which are summarized in Table 3. With regard to dopaminergic medications, to date, no randomized controlled trials have been performed to evaluate the use of levodopa to relieve psychosis in DLB, given the known side effects and the potential risk of worsening hallucinations and sleep disturbances [3]. For the same reasons, only a limited number of studies have investigated the use of antipsychotics in DLB patients, with one study reporting partial or complete amelioration of psychosis in 90% of participants after the administration of quetiapine [69], with only mild worsening in 27% of the cases of parkinsonian symptoms, while another one showed diminution of delusions and hallucinations after treatment with olanzapine [70].

Lastly, we need to bear in mind that the pathogenesis of psychosis in DLB is mainly related to right, and not left, hemispheric abnormalities, something which has been postulated after various SPECT and PET-related imaging studies [53,71,72]. Nonetheless, a left hemispheric hypoperfusion in the hippocampus, insula, ventral striatum and prefrontal, parietal and occipital cortices has also been previously linked with the presence of delusions and visual hallucinations in DLB [53].

### Brain MHPG levels are significantly reduced in DLB as opposed to AD

DLB + D patients had significantly decreased brain MHPG levels compared to the AD + D and/or AD-D group(s) in almost eight of eleven brain regions. The most remarkable decrease was noticed in BA9 and BA10, BA22 and especially thalamus. Surprisingly, in BA17, MHPG levels were significantly increased in contrast with AD patients (Figure 1).

Reduced NA concentrations in the putamen, as well as frontal, temporal and occipital cortices of DLB patients compared to AD and control subjects have been previously observed by Ohara *et al*. [54], although no MHPG levels were measured and only five patients per group were included. Accordingly, MHPG and NA levels across the cortex of AD subjects have been reported to be significantly increased and decreased, respectively, compared to control values [55]. However, the only study that comes close in comparing brain MHPG levels between DLB and AD patients, is that from Langlais *et al*. [56], in which lower MHPG and NA levels in the caudate nucleus, putamen and BA8 (frontal cortex) of LB variant AD cases compared to ‘pure’ AD cases and control subjects were observed.

The importance of MHPG in DLB as opposed to AD has been emphasized before by Herbert *et al*. [73], who confirmed that the addition of MHPG to CSF amyloid beta_1–42_ (Aβ_1–42_), total- and phosphorylated tau protein improves the discrimination of DLB from AD but not from vascular dementia. These authors also measured CSF levels of MHPG, HVA and 5-HIAA and found that not only MHPG, but also HVA, as well as 5-HIAA were significantly lower in DLB compared to AD patients, which is very similar to our results (Table 2) [74]. Given the neuropathological hallmark of substantial LC neurodegeneration in DLB [75], our results underpin the hypothesis that MHPG might indeed have an important added biomarker value to effectively distinguish between both neurodegenerative conditions. Noteworthy, in our study, MHPG levels in the LC did not significantly differ, although NA levels and MHPG/NA ratios were significantly decreased and increased, respectively, in DLB compared to both AD subgroups. Results possibly demonstrate that the presence of LB in addition to plaques and tangles might further affect the noradrenergic neurotransmission in the LC. Moreover, in AD, compensatory noradrenergic changes in response to the significant noradrenergic cell body loss in the LC have been suggested [75]. Overall, both counteracting pathophysiological processes may have resulted in seemingly unaltered MHPG levels.

Finally, the surprisingly higher MHPG levels in BA17 of DLB versus AD patients may be indicative of certain distinct monoaminergic neurotransmitter alterations in the visual association cortex. Functional deficits of such a distinguishable cerebral network have been linked to the presence of psychosis in DLB before [53,76]. However, this assumption necessitates further investigation, certainly regarding its monoaminergic etiology.

## Conclusions

By and large, we conclude that monoaminergic neurotransmitter alterations contribute differently to the neurochemical pathophysiology of depression and psychosis in DLB compared to AD. More specifically, our results support the hypothesis of a dysfunctional, decreased serotonergic neurotransmission as the main monoaminergic etiology of depression in DLB. The serotonergic pathways could potentially be even more impaired in DLB + D compared to those in AD + D, although the 5-HT deficiency theory may serve as a common feature of both. Moreover, given the generally decreased 5-HT and 5-HIAA levels, as well as 5-HIAA/5-HT turnover ratios, measured in our study, combined with the formerly suggested counteracting mechanisms of upregulated postsynaptic 5-HT_1A_ receptors and relatively preserved 5-HTT reuptake sites, a concurrent pharmacological treatment using an SSRI and a 5-HT_1A_ receptor antagonist might indeed effectively alleviate depressive symptoms in DLB patients [25]. Correspondingly, we point at a severely impaired thalamic dopaminergic neurotransmission which, supplemented with a deficient nigrostriatal dopaminergic innervation, might mediate non-parkinsonian symptoms, such as depression, in DLB as well. Furthermore, we suggest that a generally increased dopaminergic neurotransmitter activity in the prefrontal, temporal and mesolimbic cortices as well as LC and hippocampus could closely relate to psychosis in DLB. On the contrary, in AD, a decreased dopaminergic neurotransmission and increased dopaminergic catabolism, specifically in the amgydala, might function as a monoaminergic substrate of psychosis. The complexity of an altered coupling between serotonergic and dopaminergic pathways might, additionally, also account differently for the presence of psychosis in DLB compared to that in AD. On the whole, evidence is accumulating that future pharmacological treatments may target both serotonergic and dopaminergic systems to reduce these specific NPS. Finally, significantly lower MHPG levels across the brain, with the exception of BA17, in DLB compared to AD patients empathically stress its added CSF biomarker value to the traditional Aβ_1–42_ and tau proteins, as was postulated before [73].

As for future studies, the functional importance of the basal ganglia and nigrostriatal pathways in non-motor symptoms in DLB necessitate further examination, given their monoaminergic relationship with depression, among others. For example, a neurochemical analysis of the entire neostriatum, subthalamic nucleus, SN, thalamus, midbrain and cerebral cortex might create a better perspective on certain dysfunctional monoaminergic patterns which could contribute to NPS in DLB. With regard to psychosis in particular, inclusion of the right hemisphere is advised. Likewise, the relationship between NPS and acetylcholinergic neurotransmitter abnormalities in DLB as opposed to AD needs to be scrutinized as well.

## Abbreviations

5-HIAA: 5-hydroxy-3-indoleacetic acid
5-HT: 5-hydroxytryptamine (serotonin)
5-HTT: serotonin transporter
A: adrenaline
AD: Alzheimer’s disease
AD + D/-D: AD patients with/without depression
AD + D + P: psychotic AD patients with depression
AD-D-P: nonpsychotic AD patients without depression
Aβ: amyloid beta
BA: Brodmann area
Behave-AD: Behavioral Pathology in Alzheimer’s Disease Rating Scale
CMAI: Cohen-Mansfield Agitation Inventory
CSDD: Cornell Scale for Depression in Dementia
CSF: cerebrospinal fluid
DA: dopamine
DAT: dopamine transporter
DLB: Dementia with Lewy bodies
DLB + D/-D: DLB patients with/without depression
DLB + D + P: psychotic DLB patients with depression
DLB + D-P: nonpsychotic DLB patients with depression
DOPAC: 3,4-dihydroxyphenylacetic acid
GDS: Global Deterioration Scale
HE: hematoxylin & eosin
HVA: homovanillic acid
LB: Lewy bodies
LC: locus coeruleus
MFS: Middelheim Frontality Score
MHPG: 3-methoxy-4-hydroxyphenylglycol
MMSE: Mini-mental State Examination
NA: noradrenaline
NPS: neuropsychiatric symptoms
PDD: Parkinson’s disease dementia
RP-HPLC-ECD: reversed-phase high-performance liquid chromatography with electrochemical detection
SN: substantia nigra
SSRI: selective serotonin reuptake inhibitor
